# Reintroduction of the invasive mosquito species *Aedes albopictus* in Belgium in July 2013

**DOI:** 10.1051/parasite/2013054

**Published:** 2013-12-12

**Authors:** Slimane Boukraa, Fara N. Raharimalala, Jean-Yves Zimmer, Francis Schaffner, Thomas Bawin, Eric Haubruge, Frédéric Francis

**Affiliations:** 1 Gembloux Agro-Bio Tech, University of Liège 5030 Gembloux Belgium; 2 Medical Entomology Unit, Pasteur Institute Antananarivo Madagascar; 3 National Centre for Vector Entomology, Institute of Parasitology, University of Zurich Switzerland

**Keywords:** *Aedes albopictus*, Belgium, Used tire

## Abstract

Since its first report in 2000, the invasive mosquito *Aedes albopictus* was not found any more during the different entomological inspections performed at its place of introduction in Belgium between 2001 and 2012. In July 2013, one adult male was captured at the same site (a platform of imported used tires located in Vrasene, Oost-Vlaanderen Province), during a monitoring using CO_2_-baited trap. This finding suggests the reintroduction of the species in Belgium via the used tire trade.

## Abstract (fr)

Depuis sa première observation en 2000, le moustique invasif *Aedes albopictus* n’a plus été trouvé au cours des différentes inspections entomologiques réalisées en Belgique sur le site d’introduction entre 2001 et 2012. En Juillet 2013, un adulte mâle a été capturé sur ce même site (une plateforme de pneus usagés importés située à Vrasene, Province de Flandre-Orientale), durant une surveillance ayant recours à un piège à CO_2_. Cette redécouverte suggère la réintroduction de cette espèce en Belgique via le commerce de pneus usagés.

## Introduction

Emerging arbovirosis occurrences are related to a change in pathogens and/or arthropod vector distributions [10]. Environment and climate change, as well as globalization of international trade can affect these distributions [6]. Since the late 1970s, the Tiger mosquito *Aedes* (*Stegomyia*) *albopictus* (Skuse, 1894) [14] shows an explosive worldwide spreading, being currently the most invasive mosquito in the world. In Europe, it has been reported from 20 countries and is nowadays well established in the Mediterranean region [8]. *Aedes albopictus* belongs to the most important arbovirus vectors, in particular for chikungunya and dengue viruses [8]. The risk for emergence and spread of these arboviruses to no-epidemic regions has increased especially in regions where *Ae. albopictus* has established, as demonstrated by recent local transmissions of chikungunya and dengue in Croatia and France [8]. In Belgium, *Ae. albopictus* was reported for the first time in 2000 from Vrasene (Oost-Vlaanderen Province), on an used tire storage of a recycling company that imports from the USA and Japan, among other countries [11]. Several other inspections were performed after that report but no additional specimens have been found in Belgium, although the site where it has been reported from has been monitored [15, F. Schaffner unpublished data]. Thus, the species is considered as not established in Belgium and, as no control measures have been applied, it is assumed that the introduced population was naturally eliminated. Besides, there is no evidence of any additional introduction at other points of entries [15]. During this last decade, two other Asian mosquitoes were recorded in Belgium. *Ochlerotatus japonicus japonicus* (Theobald, 1901) was introduced and has established in southern Belgium (Hamois, Namur province) and *Oc*. *koreicus* (Edwards, 1917) was collected in Eastern Belgium (Maasmechelen, Limburg province), where it successfully established as well [15].

## Material and methods

In the frame of a study of bacterial endosymbionts in Belgian mosquitoes, adult mosquitoes were regularly trapped at several places throughout the country by CO_2_-baited traps Mosquito Magnet Liberty Plus^®^ (MMLP) and collected by sweep netting, and immatures were collected by the dipping method. For the present study, the surveys were conducted in two storage center of used tires recycling companies located in Oost-Vlaanderen Province. Study site A was at Vrasene (51°12′49″ N, 4°11′37″ E; 5 m above sea level) and at less of 10 km from port of Antwerp. This platform of imported used tires was regularly inspected following the find of *Ae. albopictus* in 2000 by Schaffner et al. [11]. Study site B is a company for recycling tires of local origin, located in Lochristi (51°06′18″ N, 3°52′12″ E; 5 m above sea level) and approximately 20 km southwest of the first study site. Identification of *Ae. albopictus* was first performed by morphology and then confirmed by molecular tools. The mitochondrial cytochrome oxydase subunit I (COI) was amplified [9] using the primers CI-J-1632 and CI-N-2191 [7] and sequenced. The nucleotide sequence is deposited in GenBank under accession number KF657725. Blast analysis was used to compare the obtained COI sequence with data available in NCBI (www.ncbi.nlm.nih.gov).

## Results and discussion

Early July 2013, one adult male of *Ae. albopictus* was trapped (MMLP) in Vrasene at the same used tire recycling company where it was observed in 2000. The obtained sequence compared with data available in NCBI showed 99% of similarity with those of *Ae. albopictus* from the United States and Germany. Other species collected together with *Ae. albopictus* were *Anopheles plumbeus* (Stephens, 1828), *Culex pipiens* (Linnaeus, 1758) s.l., *Cx*. *torrentium* (Martini, 1925), *Coquillettidia richiardii* (Ficalbi, 1899), and *Oc*. *geniculatus* (Olivier, 1791) (Table 1).

**Table 1.** Adult and larval mosquitoes collected in Oost-Vlaanderen province, Belgium (02-07 VII 2013).

Site	Collection method / place	*Anopheles plumbeus*	*Aedes albopictus*	*Culex pipiens s.l.*	Culex torrentium*	*Coquillettidia richiardii*	*Ochlerotatus geniculatus*	*Ochlerotatus dorsalis*
Vrasene	MMLP / TSOG	458 F	1 M	10 F	0	1 F	61 F, 20 M	0
	Sweep net / TSOG	28 F	0	0	0	0	6 F	0
	Dipper / TSS	0	0	503 L	5 L	0	36 L	0
	Dipper / TSOG	428 L	0	638 L	1925 L	0	0	0
Lochristi	MMLP / TSI	0	0	65 F	0	0	0	1 M
	Dipper / TSS	75 L	0	1424 L	0	0	0	0

L: larva; M: male; F: female; MMLP: Mosquito Magnet Liberty Plus; TSS: Tires stored in shelters; TSI: Tires stored inside; TSOG: Tires stored outside, bordering grassland; *Identification of this species based on male genitalia after emerging in laboratory.

This rediscovery of *Ae. albopictus* together with the absence of any finding during previous years (2001–2012) suggests its reintroduction into Belgium via the used tire trade. In addition, given the information about the origin of used tires recently imported by the company, the source of the species reintroduction is possibly the United States. *Aedes albopictus* is a confirmed efficient vector of Dengue and Chikungunya viruses [8], and Belgium regularly registers imported chikungunya cases [2]. Thus, if established in the country, *Ae. albopictus* may become a substantial threat to public health. A study of the survival and dispersal of this mosquito in Belgium, as well as of its bioecology in neighboring countries might provide important insights to further elucidate its invasiveness, and identify high-risk areas for mosquito proliferation and pathogen transmission. A rapid proactive response is critical for vector management because of the possibility of its establishment, according to several models [3, 4]. This includes rapid implementation of control measures, before elimination is impossible [12]. Several countries in Western Europe recently confirmed repeated introductions of *Ae. albopictus* [1, 5, 13], and thus we suggest broader and more thorough entomological surveys at the European scale to survey introduction pathways and prevent establishment, and subsequently to reduce the risks of future arbovirus transmission.
